# Crystal structures of *Burkholderia cenocepacia *dihydropteroate synthase in the apo-form and complexed with the product 7,8-dihydropteroate

**DOI:** 10.1186/1472-6807-11-21

**Published:** 2011-05-09

**Authors:** Rachel E Morgan, Gaëlle O Batot, Jennifer M Dement, Vincenzo A Rao, Thomas C Eadsforth, William N Hunter

**Affiliations:** 1 Division of Biological Chemistry and Drug Discovery, College of Life Sciences, University of Dundee, Dow Street, Dundee, DD1 5EH, UK

## Abstract

**Background:**

The enzyme dihydropteroate synthase (DHPS) participates in the *de novo *synthesis of folate cofactors by catalyzing the formation of 7,8-dihydropteroate from condensation of *p*-aminobenzoic acid with 6-hydroxymethyl-7,8-dihydropteroate pyrophosphate. DHPS is absent from humans, who acquire folates from diet, and has been validated as an antimicrobial therapeutic target by chemical and genetic means. The bacterium *Burkholderia cenocepacia *is an opportunistic pathogen and an infective agent of cystic fibrosis patients. The organism is highly resistant to antibiotics and there is a recognized need for the identification of new drugs against *Burkholderia *and related Gram-negative pathogens. Our characterization of the DHPS active site and interactions with the enzyme product are designed to underpin early stage drug discovery.

**Results:**

An efficient recombinant protein expression system for DHPS from *B. cenocepacia *(*Bc*DHPS) was prepared, the dimeric enzyme purified in high yield and crystallized. The structure of the apo-enzyme and the complex with the product 7,8-dihydropteroate have been determined to 2.35 Å and 1.95 Å resolution respectively in distinct orthorhombic crystal forms. The latter represents the first crystal structure of the DHPS-pterin product complex, reveals key interactions involved in ligand binding, and reinforces data generated by other structural studies. Comparisons with orthologues identify plasticity near the substrate-binding pocket and in particular a range of loop conformations that contribute to the architecture of the DHPS active site. These structural data provide a foundation for hit discovery. An intriguing observation, an artifact of the analysis, that of a potential sulfenamide bond within the ligand complex structure is mentioned.

**Conclusion:**

Structural similarities between *Bc*DHPS and orthologues from other Gram-negative species are evident as expected on the basis of a high level of sequence identity. The presence of 7,8-dihydropteroate in the binding site provides details about ligand recognition by the enzyme and the different states of the enzyme allow us to visualize distinct conformational states of loops adjacent to the active site. Improved drugs to combat infections by *Burkholderia sp. *and related Gram-negative bacteria are sought and our study now provides templates to assist that process and allow us to discuss new ways of inhibiting DHPS.

## Background

**Figure 1:**
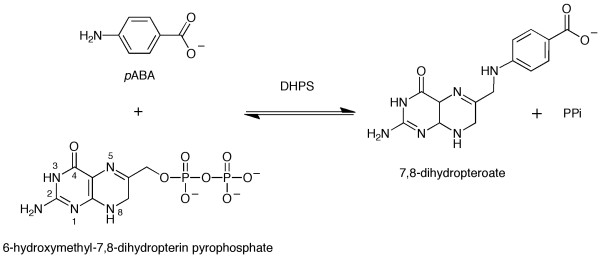
**The reaction catalyzed by dihydropteroate synthase**. 7,8-dihydropterin pyrophosphate reacts with *p*-aminobenzoic acid (*p*-ABA) to yield 7,8-dihydropteroate and pyrophosphate.

Dihydropteroate synthase (DHPS, EC: 2.5.1.15) catalyses the reaction of 6-hydroxymethyl-7,8-dihydropterin-pyrophosphate with *p*-aminobenzoic acid (*p*-ABA) to yield 7,8-dihydropteroate and pyrophosphate (Figure 1). In so doing the enzyme supports the biosynthesis of folate, a key metabolite required to support the synthesis of DNA, and proteins. The provision of folates, either by synthesis in plants and microorganisms or as acquired in the diet by mammals, supports life. The folate biosynthetic pathway is absent from humans and contains several highly valued drug targets for treatments of numerous infections [1,2]. The folate pathway consists of a number of enzymes in addition to DHPS, including: 6-hydroxymethyl-7,8-dihydropterin pyrophosphokinase (HPPK), dihydrofolate synthetase (DHFS) and dihydrofolate reductase (DHFR). Drugs that inhibit DHFR and DHPS are used in the treatment of infections by the apicomplexan parasites *Plasmodium sp. *and *Toxoplasma gondii *[2-4]. In these species DHPS is part of a bifunctional enzyme fused to HPPK [5].

The Gram-negative aerobic bacterium *Burkholderia cenocepacia *is an opportunistic pathogen and a member of the *Burkholderia *complex, a closely related group of bacteria, which cause particular problems for cystic fibrosis patients [6]. Other members of the genus are also pathogenic; *B. pseudomallei *is the causal agent for melioidosis [7], a serious infection found primarily in South East Asia, and *B. mallei *is responsible for glanders, an infection of livestock [8]. A characteristic of *Burkholderia *species, and one that makes them particularly troublesome pathogens is that they are highly resistant to a wide range of antibiotics [9-12].

DHPS is a validated drug target for the treatment of diseases caused by bacteria and protozoan parasites [1,2]. Sulfonamides in particular are inhibitors of this enzyme and are used as antibiotics [13]. However, increasing levels of resistance to sulfonamides has been observed and there is a need for new drugs to compensate for this [9,14-17]. The value of accurate structural information to support early stage drug discovery is well recognized [18] and characterization of the active site of DHPS from pathogenic organisms has the potential to support the design of new treatments.

Structures of DHPS from Gram-negative and Gram-positive bacteria have been reported [19-23] and also the bifunctional HPPK-DHPS from *Saccharomyces cerevisiae *and *Francisella tularensis *[24,25]. The structural studies extend to characterization of the oxidized product analogue, pteroic acid, and a series of pterin derivatives in complex with *Bacillus anthracis *DHPS (*Ba*DHPS) [21,26] and also a series of pterin derivatives, and a complex of *Escherichia coli *DHPS (*Ec*DHPS) with sulfanilamide [19]. One structure, that of *Thermus thermophilus *DHPS complexed with *p*-ABA has also been solved [Protein Data Bank (PDB) ID: 2DZA]. One observation, reported in several studies [e.g. [20]] and made by our inspection of PDB entries is the pronounced conformational flexibility of loops around the active site. This has resulted in the omission of important residues from the structural models.

Here we report the expression, purification and crystallization of *Bc*DHPS. We describe the crystal structures of the apo-enzyme, the first complex with the actual enzyme product 7,8-dihydropteroate, which produces a more complete view of the active site than most other structures, similarities and differences between DHPS structures, and discuss molecular features that might be exploited to derive novel inhibitors.

## Results and Discussion

### General comments

An efficient recombinant source of *Bc*DHPS was prepared and a purification protocol established that resulted in approximately 20 mg of pure enzyme from one litre of bacterial culture. The purified protein was used in co-crystallization screens with the inhibitor sulphadoxine or the enzyme product 7,8-dihydropteroate. Distinct orthorhombic crystal forms were obtained. However, analysis of the crystals grown in the presence of sulphadoxine revealed only water molecules in the active site and this therefore represents the apo-*Bc*DHPS structure determined to 2.35 Å resolution. The structure of *Bc*DHPS complexed with 7,8-dihydropteroate was refined to 1.95 Å resolution. Initial phases for the product complex were obtained by molecular replacement calculations using the *Ec*DHPS structure [PDB ID: 1AJ2, [19]], which has 44% amino acid sequence identity to *Bc*DHPS, as the search model. The model for molecular replacement calculations to solve the apo-structure of *Bc*DHPS was the product complex. *Bc*DHPS comprises 292 amino acids. The apo-enzyme crystallized in space group ^*C*222^_1 _with one molecule in the asymmetric unit. A number of residues could not be modeled in the electron density maps and so were omitted hence the model for this structure consists of 268 residues. The crystals for the complex structure display space group *P*2_1_2_1_2_1 _and there are two molecules, labeled A and B, in the asymmetric unit. Here also disorder was evident and several residues in each subunit are missing from the model. Subunit A consists of 280 residues and subunit B consists of 281. These subunits are similar in structure and superimpose with an r.m.s.d. of 0.34 Å (over 280 aligned Cα atoms). The two structures superimpose with an r.m.s.d. of 0.37 Å (over 268 aligned Cα atoms, superimposing subunit A of the complex structure). Such a close agreement suggests that there are no gross structural changes upon ligand binding but as will be explained, ligand binding promotes greater order in loops that contribute to the active site.

### The Overall Structure and comparison with orthologues

**Figure 2:**
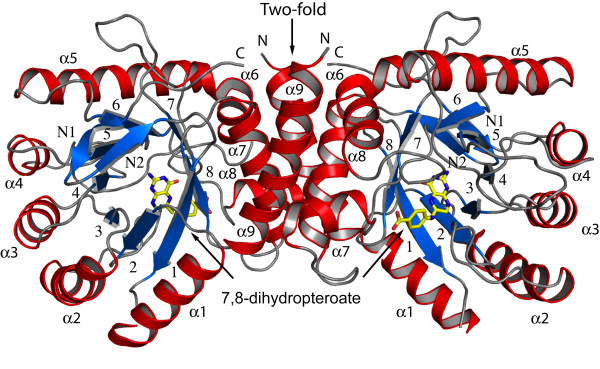
**The structure of *Bc*DHPS and *Bc*DHPS complex**. The overall structure of the *Bc*DHPS dimer with 7,8-dihydropteroate bound. α-helices are shown in red and β-sheets in blue, with C atoms of the ligand 7,8-dihydropteroate shown in yellow.

*Bc*DHPS displays a triosephosphate isomerase (TIM)-barrel fold, consisting of eight parallel β-strands surrounded by eight α-helices (Figure 2). At the N-terminus there is an antiparallel alignment of two short β-strands, termed Nβ1 and Nβ2, linked by a tight turn. This is a feature common to some other DHPS structures. We assign β1 as the first strand of the β_8_:α_8 _barrel. Residues 234-242 form a short α-helix inserted between β7 and α8 and is considered part of loop 7. Size-exclusion gel filtration indicates that *Bc*DHPS is a dimer, of approximate mass 68 kDa, in solution (data not shown). The apo-enzyme structure has a single polypeptide chain in the asymmetric unit whilst the product complex has two. In the latter structure, chains A and B form a dimer stabilized by an interface with a surface area (~1,200 Å^2^) that is approximately 10% of that of a single subunit (~12,000 Å^2^). Residues on α9 of one subunit interact with residues on α6, α7 and α8 of the partner subunit. Nearly 40 amino acids from each subunit participate in around 20 direct hydrogen bonds and salt bridge interactions, plus numerous water mediated contacts between the subunits and The same dimer is formed in the apo-structure by the symmetry operation -x, y, -z + ½.

The alignment of the central β-strands results in the localization of the N-terminal segments of these strands at one end of the barrel and the C-terminal segments at the other. On the N-terminal side there are polypeptide segments that form loops connecting the α-helix to the β-strand that follows. These loops are relatively short and well defined in the electron density maps (data not shown). On the C-terminal side of the TIM-barrel, where the active site is located, there are longer, more flexible loops, called the C-terminal loops, that link a β-strand with the following α-helix. The shorter N-terminal loops of TIM-barrel structures are suggested to contribute stability of the protein fold whereas the C-terminal loops contribute to enzyme activity by creation of the active site [27]. The first C-terminal loop, linking β1 to α1 could not be modeled in either structure, due to poorly defined density, suggesting a significant degree of conformational flexibility. Modeling of loop 2, linking β2 to α2 was possible only in the case of the product complex. The increased order observed for the complex is consistent with the stabilizing effects of ligand binding, as loop 2 contributes to the active site, a feature that will be discussed later.

**Figure 3:**
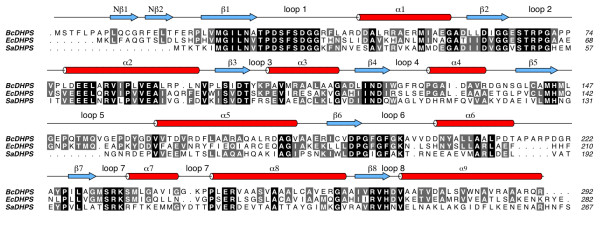
**Sequence alignment of three dihydropteroate synthases**. The amino acid sequences for DHPS from *B. cenocepacia, E. coli *and *S. aureus *are aligned. The secondary structure relating to *Bc*DHPS is shown colored as in Figure 2. Those residues shaded in black are conserved in all three sequences and grey marks conservation in two of the three.

The overall sequence identity of *Bc*DHPS with the enzyme from other Gram-negative species is in the region of 40-60% and the fold of *Bc*DHPS and dimeric quaternary structure is consistent with that observed for DHPS from other species. Structural overlays of the *Bc*DHPS subunit with orthologous structures identified greatest similarity to *E. coli *DHPS with r.m.s.d. values of 1.35-1.37 Å [PDB IDs: 1AJ0, 1AJZ and 1AJ2, values given for the complex subunit A, over 264, 264 and 263 aligned Cα atoms]. R.m.s.d. values of 2.1 Å (for both subunit A and B, over 242 (A) and 239 (B) aligned Cα atoms) were observed for DHPS structures from *Streptococcus pneumoniae *[PDB IDs: 2VEF and 2VEG] and 1.5 Å (value for subunit A, over 239 aligned Cα atoms) for the *Mycobacterium tuberculosis *DHPS structure [PDB ID: 1EYE]. The orientation of DHPS subunits with respect to each other varies [20] but there is no evidence to suggest that this has an influence on activity. The DHPS active site is formed entirely within a single subunit. The difference in subunit orientation may simply reflect sequence variation between species. The residues that occur at the dimer interface (on α6, α7, α8 and α9) show only a low level of sequence conservation (Figure 3).

**Figure 4:**
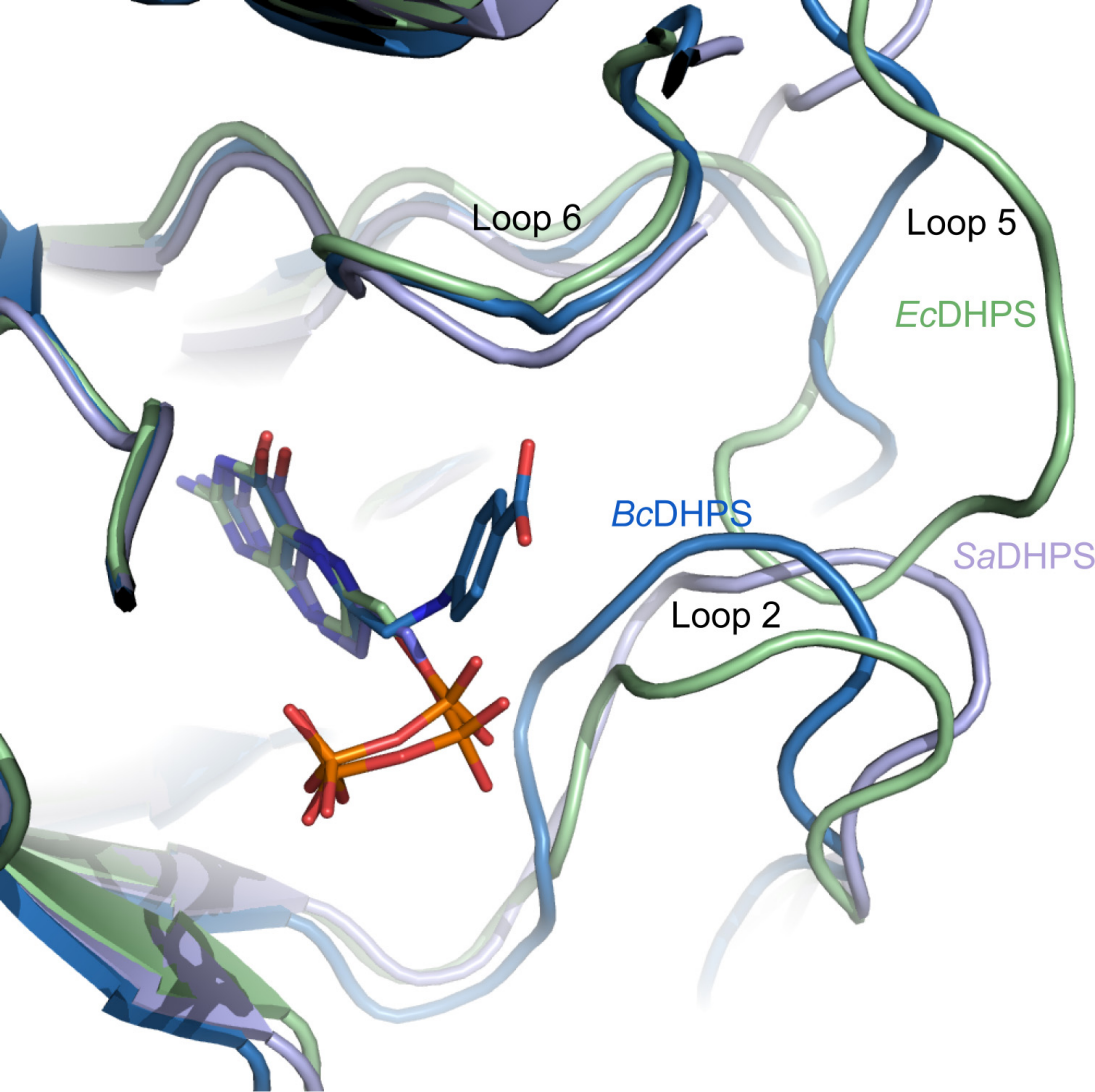
**The variations in the positions of active site loops across three DHPS structures**. Superposition of the structures of *Bc*DHPS with 7,8-dihydropteroate bound (blue), *Ec*DHPS with 7,8-dihydropterin pyrophosphate bound (green, [PDB ID: 1AJ2]) and *Sa*DHPS with 6-hydroxymethylpterin-diphosphate bound (violet, [PDB ID: 1AD4]).

The flexible C-terminal loops are the regions where the most structural variation between DHPS models has been observed. Of particular note are loops 1 and 2. The amino acid residues that comprise these loops are highly conserved in DHPS orthologues (Figure 3) but they are absent from the majority of published crystallographic models. Where the electron density maps have allowed for their inclusion it is noted that the polypeptides display different conformations. This points to a high propensity to disorder due to flexibility and an important role in enzyme function. One role for these loops has been proposed, namely that they close over and shield the active site from bulk solvent during catalysis [20]. To this we would add a potentially important thermodynamic contribution to the enzyme function. Changes in conformational entropy associated with distinct configurations of the loop structures might contribute to the free energy of protein-ligand association [28]. We note the presence of conserved glycine residues at the C-terminal end of β1 and on loop 2 itself (Figure 3) that may contribute to the flexibility in this part of the structure. Loop 2 is closer than loop 1 to the enzyme active site and adjacent to loop 5 (Figure 4). The residues that constitute loop 5 are less well conserved (Figure 3). *Bc*DHPS has 17 residues in this loop, a similar number, 16, are present in the *E. coli *enzyme, whilst *Staphylococcus aureus *DHPS has only 9 residues in the loop (Figure 3). This lack of conservation in loop 5 and interactions with the conformationally labile loop 2 likely contributes to the variation observed when different DHPS structures are compared (Figure 4).

As an aside we mention the following observation made by a reviewer. At the C-terminal end of β1, leading into loop 1 the amino acid sequence in *Bc*DHPS Asn28-Ala29-Thr30. In *Ec*DHPS and *Sa*DHPS the sequence is Asn-Val-Thr. This is a well-recognized N-glycosylation signal, Asn-X-Ser/Thr [29]. However, in each case DHPS is a cytosolic bacterial enzyme from organisms lacking in glycosides.

### Binding of 7,8-dihydropteroate

**Figure 5:**
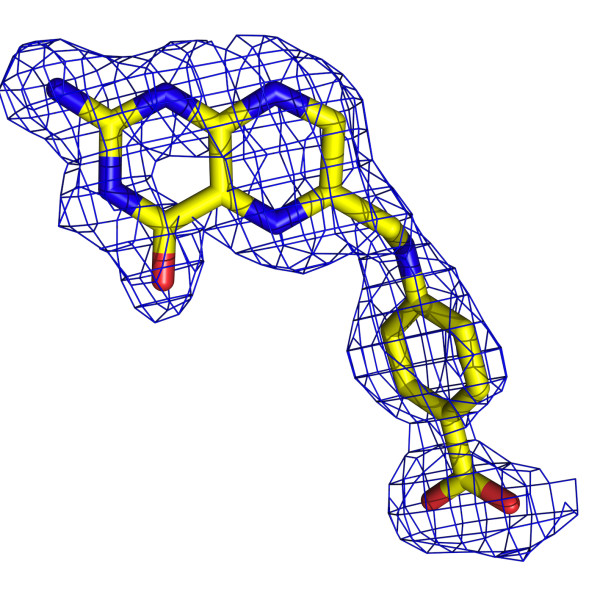
**The omit electron density associated with 7,8-dihydropteroate**. The blue chicken wire represents the Fo-Fc omit map contoured at 2.5σ. The ligand is colored according to atom type; C yellow, N blue, O red.

**Figure 6:**
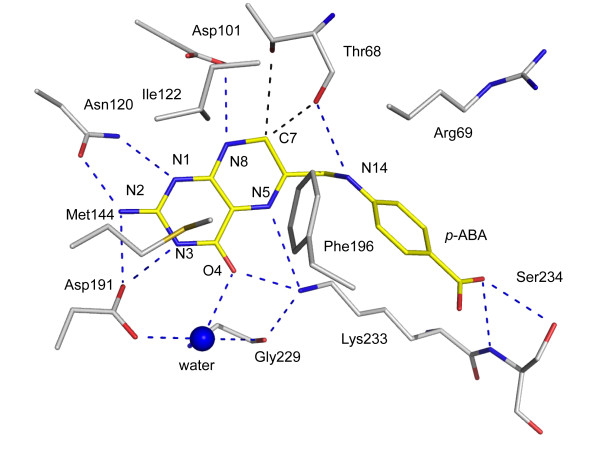
**The *Bc*DHPS binding site of 7,8-dihydropteroate**. Potential hydrogen bonds formed between N and O atoms are depicted as blue dashed lines. C-H•••O interactions involving C12 as black dashed lines. The ligand is colored as in Figure 5. C atoms of DHPS are colored gray, the S atom of Met144 yellow.

The active site of DHPS is positioned at the C-terminal end of the central β-barrel. Here, the product of the enzyme reaction, 7,8-dihydropteroate is ordered (Figure 5), placed with the pterin directed down into the barrel with the *p*-ABA moiety pointing out towards the surface of the protein (Figure 2). Residues located on strands β3, β4, β6, β7 and β8 participate in interactions with the product. The interactions formed between the enzyme and the ligand are very similar in both subunits and therefore only details of subunit A are presented (Figure 6) unless stated otherwise.

The ligand possesses nine functional groups of which eight participate in direct hydrogen bonds with DHPS. Four hydrogen bond donors, N2, N3, N8 and N14 interact with functional groups on Asn120, Asp191, Asp101 and Thr68 respectively. The N1 atom of the ligand accepts a hydrogen bond donated by Asn120 ND2. On the other side of the pterin, O4 and N5 accept hydrogen bonds donated from Lys233 NZ. This lysine side chain is also held in place by a hydrogen bond donated to the carbonyl oxygen of Gly229. An ordered water molecule interacts with O4 and Asp191 OD1. A carboxylate oxygen, on the *p*-ABA moiety, accepts hydrogen bonds donated from the main chain amide and side chain hydroxyl groups of Ser234. In subunit A the other oxygen interacts with two water molecules that in turn interact with the carbonyl and amide groups of Gly195 and Met235 respectively (data not shown). In subunit B only the latter interaction is observed. The proximity of C7 to the carbonyl oxygen and OG1 hydroxyl of Thr68, distances range from 3.1 Å to 3.5 Å in subunits A and B, indicates the potential for C-H•••O hydrogen bonds. These are weak but not insignificant stabilizing interactions [30]. The *E. coli *DHPS structure [PDB ID: 1AJ2] with 7,8-dihydropterin pyrophosphate bound in the active site, shows Thr62 (the equivalent of Thr68 in *Bc*DHPS) interacting with the phosphate groups of the ligand [19]. In our *Bc*DHPS structure Thr68, part of loop 2, interacts with Asp101 and the dihydropterin. This suggests that the conformation of loop 2 alters depending on which ligand is bound in the active site.

The pterin is sandwiched between a cluster of hydrophobic residues (Ile122, Met144 and Phe196) on one side (Figure 6) and the guanidinium group of Arg268 on the other (data not shown). A salt-bridge interaction between Arg268 and Asp62 helps to determine the structure in this part of the active site and Asp62 also interacts with the side chain of Asn120 (data not shown).

The structure of *Ba*DHPS in complex with pteroic acid [21] shares most of the key interactions described above. A notable difference between pteroic acid and 7,8-dihydropteroate is that N8 in the latter is a hydrogen bond donor, in the former a hydrogen bond acceptor. The distances between N8 and the carboxylate of the conserved Asp101 in both structures are close to 3.2 Å. This suggests to us that in the *Ba*DHPS ligand complex Asp101 is likely protonated.

Sulfonamides, which are used to treat infections by *Burkholderia *[31], inhibit DHPS by binding in the *p*-ABA binding site, as shown through competition, resistance and structural data [19,20,22]. The complex of *Ec*DHPS with sulfanilamide showed the ligand positioned between Lys221 and Arg63. This is similar to the placement of the *p*-ABA groups in the pteroic acid and 7,8-dihydropteroate complexes, which are near to loop 2. Mutations in residues that contribute to loop 2 have been noted to correlate with resistance to sulfonamide and dapsone; in particular the equivalent residues to Thr68 in *Bc*DHPS [19,20]. The alignment of three bacterial DHPS sequences (Figure 3) shows that positions equivalent to 234 in our structure are occupied either with serine or arginine. Given that Ser234 interacts with the *p*-ABA moiety the difference between serine and arginine has interesting implications regarding differences in ligand binding sites across species.

The availability of high-resolution crystal structures offers opportunities to exploit structure-based methods to derive information on DHPS inhibitors that would support early stage drug discovery. The pterin binding pocket of DHPS is highly conserved across species. In the enzyme:product complex for *Bc*DHPS we described the contributions of 14 amino acids above; ten of these residues are strictly conserved (Figure 3) and two, Gly229 and Met235, only use main chain functional groups to bind the 7,8-dihydropteroate. The structures clearly reveal the necessity for a planar entity and the arrangement of hydrogen bond donors and acceptors required to optimize interactions in the pterin binding pocket. The presence of an ordered water molecule mediating interactions between the ligand the enzyme suggests a position where a new hydrogen bonding interaction might be sought. Unfortunately the disorder (flexibility) of loop 2 means that many of the crystal structures have limitations with respect to drug design. We were fortunate that the product complex of *Bc*DHPS allowed us to model loop 2 and the complete structure we obtained provides an improved template suited for *in silico *screening. We recognize that there is flexibility around the edge of the active site part of the binding site and that this flexibility might make an important thermodynamic contribution to DHPS function. This observation suggests that ligands able to interact with and reduce conformational freedom of loop 2 have the potential to increase the free energy of ligand binding, as might substrate, and these could provide suitable chemical matter for future development.

#### A Potential Cys-Arg Sulfenamide Bond

**Figure 7:**
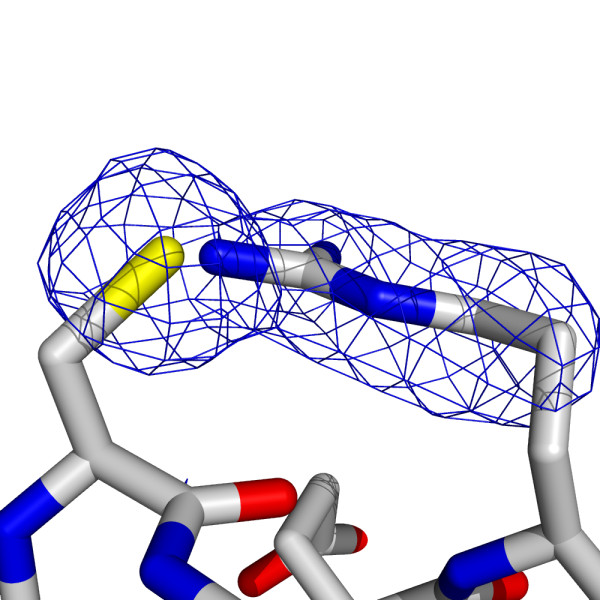
**The positions of Cys258 and Arg262 in the product complex**. The blue chicken wire represents the Fo-Fc omit map contoured at 3.5σ.

**Figure 8:**
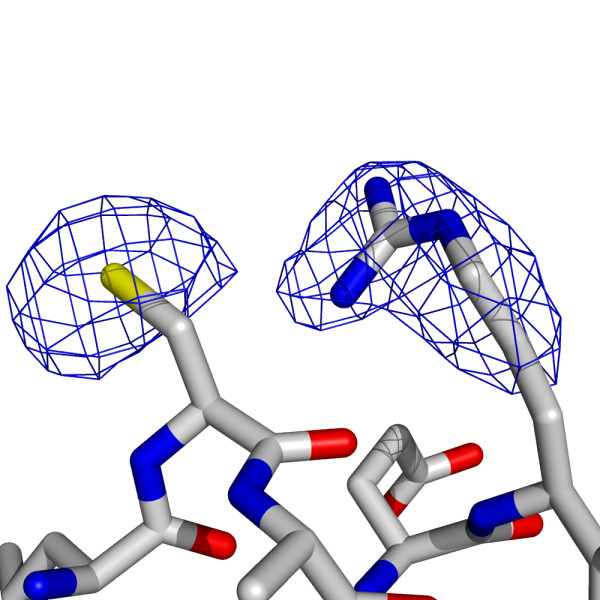
**The positions of Cys258 and Arg262 in apo-*Bc*DHPS**. The blue chicken wire represents the Fo-Fc omit map contoured at 3.5σ.

During refinement of the *Bc*DHPS:dihydropteroate complex we noted an unusual feature in, first the electron density surrounding the side chains of Cys258 and Arg262, and then in omit difference maps that is worthy of mention. The density suggested the possibility of a covalent linkage, possibly a sulfenamide bond between Cys258 SG and Arg262 NH2 (Figure 7). The crystallographic analysis of the apo-enzyme clearly showed the normal side chains not interacting with each other (Figure 8). Our first assessment was that conformational flexibility had produced a mixture of rotamers that were overlapping. However, there is no evidence of an alternative rotamer for either residue. We were surprised that the feature was conserved in each subunit of the asymmetric unit and sought data to identify if an unusual covalent linkage was present. The analysis of freshly purified protein solution using Fourier Transform Ion Cyclotron Resonance mass spectrometry and Top-Down fragmentation failed to find any evidence for an unusual S-N covalent linkage. Similarly analysis of DHPS crystals, from the same batch of protein that gave the complex structure, by trypsin cleavage followed by mass spectrometry did not find any evidence to substantiate S-N bond formation.

These residues are located towards the C-terminal end of α8, at the surface of the protein and contribute to the dimer interface. The residues are not conserved in orthologues, and any interaction is unlikely to be physiologically relevant. A covalent modification may have occurred due to the experimental conditions in this particular instance and would likely have followed from oxidation of the cysteine.

## Conclusion

The structure of *B. cenocepacia *DHPS has been solved in the presence and absence of a product from the reaction that it catalyses, 7,8-dihydropteroate. These structures closely resemble published DHPS structures from other species. Loop 2 has been modeled in the *Bc*DHPS product complex. This loop, which is highly conserved in terms of amino acid sequence and inherently flexible, makes important contributions to the creation of the active site. The structure of the *Bc*DHPS:7,8-dihydropteroate complex, with loop 2 modeled provides a useful template to aid the design of new inhibitors of this enzyme.

## Methods

### Protein expression and purification

The gene encoding *Bc*DHPS [UniProtKB Q1BXC8] was amplified from genomic DNA (Belgian Co-ordinated Collections of Micro-organisms, bacteria collection (BCCM/LMG)) using 5'-**CATATG**TCCCCGTTCCTTCCCGC-3' and 5'-**GGATCC**TTATCGTTGCCGCGCGGCT-3' as the forward and reverse primers respectively (Thermo Scientific). The restriction sites for *Nde*I and *Bam*HI are in bold. The PCR product was ligated into pCR2.1-TOPO using the TOPO^® ^TA Cloning^® ^Kit (invitrogen) and cloned into a modified pET15b (Novagen) expression vector. This vector produces the protein with a N-terminal hexa-His tag with a Tobacco Etch Virus (TEV) protease cleavage site. DNA sequencing confirmed the identity of the construct and the vector was heat-shock transformed into *E. coli *BL21 (DE3) cells for expression.

Cells were grown at 37°C in 1 L of Luria-Bertani media supplemented with carbenicillin (50 μgL^-1^). Gene expression was induced, once an OD_600 _of 0.6 was reached, by addition of 1 mM isopropyl-β-D-thiogalactopyranoside. The culture was maintained for a further 16 hours at 22°C. Cells were harvested by centrifugation at 3,500 g for 30 min at 4°C.

The cells were resuspended into a lysis buffer (20 mL, 50 mM Tris-HCl, pH 7.5, 250 mM NaCl, 25 mM imidazole) containing DNAse I (0.2 mg) and two tablets of a cocktail of EDTA-free protease inhibitors (Roche) and lysed using a French press at 16,000 psi. The resultant lysate was centrifuged at 35,000 g for 30 min at 4°C and the supernatant loaded onto a pre-equilibrated HisTrap HP 5 mL column (GE Healthcare) pre-charged with Ni^2+^. A linear gradient of imidazole, 25 mM to 1 M was applied to elute *Bc*DHPS and the fractions were analyzed by sodium dodecyl sulfate polyacrylamide gel electrophoresis. The *Bc*DHPS containing samples were pooled and incubated with His-tagged TEV protease (2 mg) at 30°C for 3 hours and then dialyzed into 50 mM Tris-HCl, pH 7.5, 250 mM NaCl. The protein was loaded onto a HisTrap HP 5 ml column and eluted as described above, untagged *Bc*DHPS did not bind to the column. A Superdex 200 26/60 size exclusion column (GE Healthcare) equilibrated with 50 mM Tris-HCl, 250 mM NaCl pH 7.5 was used to further purify the protein. This column had previously been calibrated with molecular weight standards, blue dextran (>2,000 kDa), thyroglobulin (669 kDa), ferritin (440 kDa), aldolase (158 kDa), conalbumin (75 kDa), ovalbumin (43 kDa), carbonic anhydrase (29.5 kDa), ribonuclease A (13.7 kDa) and aprotinin (6.5 kDa); (GE Healthcare; data not shown).

The protein eluted with one peak of a mass of approximately 68 kDa corresponding to a dimer. Fractions containing the protein were pooled and concentrated using Amicon Ultra devices (Millipore) to 30 mg L^-1 ^for subsequent use. The purity and identity of the protein was further confirmed by matrix-assisted laser desorption/ionization mass spectrometry, with a yield of approximately 20 mg L^-1 ^of cell culture.

### Crystallization and data collection

Crystallization conditions were screened using the sitting drop vapor diffusion method in 96-well plates. This was achieved with a Phoenix liquid handling system (Rigaku, Art Robins Instruments) and the commercially available screens Classics (Qiagen) and JCSG+ (Molecular Dimensions). Optimized conditions were achieved using hanging drop vapor diffusion methods with drops consisting of 1 μL protein solution and 1 μL reservoir solution.

Crystals of apo-*Bc*DHPS were obtained with reservoir conditions of 0.3 M Tris-HCl, pH8 and 10% polyethylene glycol (PEG) 8000 and protein solution with *Bc*DHPS at 7.5 mg mL^-1 ^and sulphadoxine (2 mM; Sigma-Aldrich). Rectangular crystals with approximate dimensions 0.2 mm × 0.2 mm × 0.15 mm grew in one week. A specimen was transferred to a mixture of reservoir solution and 40% PEG 400, for approximately 15 seconds, then transferred into a stream of cold nitrogen (-170°C) and a data set collected.

Crystals of the complex were obtained with reservoir conditions of 0.1 M Tris-HCl pH 8, 10% PEG 8000, 0.3 M MgCl_2_. The protein solution (7.5 mg mL^-1^) was incubated at 4°C for 4 hours with 7,8-dihydropteroate (2 mM; Schircks Laboratories). The crystals consisted of multiple plates with dimensions 0.2 mm × 0.2 mm × 0.10 mm. A fragment of a crystal, of dimensions 0.1 mm × 0.1 mm × 0.05 mm. was transferred to a mixture of the reservoir solution and 25% ethylene glycol prior to cooling. The samples diffracted well and a data set was collected.

Data for both crystals were collected in-house with a Micromax 007 rotating anode generator (copper Kα, λ = 1.5418 Å) and R-AXIS IV^++ ^dual image plate detector (Rigaku). Processing and scaling of the data was carried out using MOSFLM [32] and SCALA [33]. The apo-*Bc*DHPS crystal belonged to space group *C*222_1_, with unit cell parameters a = 73.9 Å, b = 89.43 Å, c = 87.60 Å, α = β = γ = 90 °. There is one subunit in the asymmetric unit with V_M _values of 2.3 Å^3 ^Da^-1 ^and a solvent content of approximately 50%.

The crystal of the *Bc*DHPS:7,8-dihydropteroate complex displayed space group *P*2_1_2_1_2_1_, with unit cell parameters a = 73.66 Å, b = 86.92 Å, c = 88.79Å, α = β = γ = 90 °. Two subunits in the asymmetric unit gives V_M _of 2.3 Å^3 ^Da^-1 ^and solvent content of approximately 50%. The complex structure was solved in PHASER [34] using molecular replacement with the *E. coli *DHPS structure [PDB ID: 1AJ2]. The structure of apo-*Bc*DHPS was solved using the complex structure for molecular replacement. Alteration of the model, including addition of solvent molecules was achieved in COOT [35]. The model was refined using REFMAC5 [36] with non-crystallographic symmetry (NCS) restraints in the initial rounds of refinement. Translation, libration, screw analysis (TLS) was also applied [37]. Crystallographic statistics are presented in Table 1. R.m.s.d. values comparing the previously published structures were achieved using DALI [38]. The figures were prepared with PyMOL [39]. Amino-acid sequence alignments were carried out using the program MUSCLE [40]. The analysis of the dimer interface used the PISA server [41].

**Table 1:** Crystallographic statistics

	Apo-*Bc*DHPS	*Bc*DHPS complex
**Data Collection**	^*C*222^_1_	*P*2_1_2_1_2_1_
Unit Cell (Å)	73.9 89.43 87.60	73.66 86.92 88.79
Resolution Range (Å)	57.25-2.35 (2.48-2.35)	29.60-1.95 (2.001-1.95)
Number of Reflections	84141	183983
Number of Unique Reflections	12564	41750
Completeness (%)	99.5 (97.1)	98.9 (93.6)
Multiplicity	6.7 (4.2)	4.4 (3.7)
*R*_*merge *_(%)^†^	11.7 (36.4)	5.4 (36.9)
<I/s(I)>	10.0 (2.4)	14.4 (3.1)
**Refinement**		
*R*_*work*_^‡^	0.208	0.176
*R*_*free*_^§^	0.274	0.211
r.m.s.d. from ideal values, bond lengths (Å)	0.0083	0.0109
r.m.s.d. from ideal values, bond angles (°)	2.1597	1.2320
DPI* (Å)	0.3917	0.1496
**Ramachandran analysis**		
Residues in favored regions (%)	95.4	96.7
Residues in allowed regions (%)	0.4	1.3
**Model Statistics**		
Wilson *B*-factor (Å^2^)	47.7	27.4
Protein residues:		
No. in subunit A & B	268	280 281
*B*-factor A & B (Å^2^)	39.5	27.8 30.4
Additional groups:		
Ligand *B*-factor in subunit A & B	NA	31.4 33.1
Water No./*B*-factor	89/35.9	324/21.9
Ethylene glycol No./*B*-factor	4/35.5	4/38.7
Chloride No./*B*-factor	NA	1/34.3

Values in parentheses refer to the highest resolution shell. ^†^*R*_*merge *_= ∑_*hkl*_*∑*_*i*_*|*I_*i*_(*hkl*) - <I(*hkl*)>*|/ *∑_*hkl*_*∑*_*i *_I_*i*_(*hkl*); where I_*i*_(*hkl*) is the intensity of the *i*th measurement of reflection *hkl *and <I(*hkl*)> is the mean value of I_*i*_(*hkl*) for all *i *measurements. ^‡^*R*_*work *_= ∑_*hkl*_||*F*_*o*_|-|*F*_*c*_||/∑|*F*_*o*_|, where *F*_*o *_is the observed structure factor and *F*_*c *_is the calculated structure factor. ^§^*R*_*free *_is the same as *R*_*cryst *_except calculated with a subset, 5%, of data that are excluded from refinement calculations. *The diffraction-component precision index as defined by Cruickshank [42].

### Protein Data Bank accession numbers

Coordinates and structure factors have been deposited with accession code 2Y5J (apo-enzyme) and 2Y5S (product complex).

## Abbreviations

E.C.: Enzyme Commission; HPPK: 6-hydroxymethyl-7,8-dihydropterin pyrophosphokinase; PEG: polyethylene glycol; PDB: Protein Data Bank; r.m.s.d.: root mean square deviation; DHPS: dihydropteroate synthase TEV: Tobacco Etch Virus; TLS: Translation/Libration/Screw.

## Authors' contributions

REM collected data, solved and refined the structure of the complex, completed refinement of the apo-structure. Investigated the Cys-Arg bond and drafted the manuscript. GOB cloned, expressed, purified and crystallized the DHPS complex. JMD crystallized apo-DHPS, collected data and contributed to the refinement. VAR prepared the expression system. TCE aided the refinement of the complex structure. WNH conceived of the study, contributed to data analysis and checking, and manuscript completion. All authors read and approved the final manuscript.
